# Safety Profiles of Polymyxins, Aminoglycosides, and Imipenem/Cilastatin/Relebactam (IMI/REL) in the Treatment of Gram-Negative Infections: A Literature Review

**DOI:** 10.3390/antibiotics15050422

**Published:** 2026-04-22

**Authors:** Hannah Collings, Medi Stone, Anouska Jha, François-Xavier Houde, Florence D’Adamo, Todd Waldenberg, Emre Yücel

**Affiliations:** 1 Adelphi Values PROVE, Bollington SK10 5JB, UK; 2 Merck Canada Inc., Kirkland, QC H9H 4M7, Canada; 3 Merck & Co., Inc., Rahway, NJ 07065, USA

**Keywords:** gram-negative infections, polymyxins, aminoglycosides, adverse events, safety, imipenem/cilastatin/relebactam, mortality

## Abstract

**Background/objectives:** Gram-negative bacterial infections are associated with significant morbidity and mortality. This targeted literature review (TLR) aimed to descriptively synthesise safety outcomes reported for polymyxins, aminoglycosides, and imipenem/cilastatin/relebactum (IMI/REL) in adult patients with Gram-negative infections. **Methods:** A TLR was conducted to identify published literature from 2015 to 2025. A database search was conducted on 14 February 2025, using the OVID^®^ platform and grey literature search reviewed publications from the European Congress of Clinical Microbiology and Infectious Diseases (ECCMID) and Infectious Disease (ID) Week. Safety outcomes included nephrotoxicity, other toxicities (e.g., haematological, hepatological), renal impairment, treatment-related (i.e., explicitly related to the antimicrobial treatment or its complications) discontinuation, and treatment-related mortality. **Results:** Sixty-eight publications were included. Definitions of nephrotoxicity varied between publications; RIFLE and KDIGO classifications were typically used. Definitions of renal impairment included renal risk/injury/failure and acute kidney injury (AKI). Colistin (*n* = 63) showed nephrotoxicity rates of 30.8–56.4%; renal impairment of 15.0–53.8%; treatment-related discontinuation of 12.5–23.0%; and treatment-related mortality from 20.0 to 39.1%. Polymyxin B showed nephrotoxicity rates of 14.6–54.9%; renal impairment rates ranging from 5.3 to 58.1%; and treatment-related mortality of 7.0% (*n* = 1). Aminoglycoside data were limited (*n* = 2) but showed nephrotoxicity rates of 77.8% and renal impairment of 18.8%. IMI/REL (*n* = 6) demonstrated nephrotoxicity of 10.3–17.2%; renal impairment of 0.0–20.7%; treatment-related discontinuation of 0.0–2.3%; and treatment-related mortality of 0.0–0.7%. **Conclusions:** Polymyxins/aminoglycosides had more frequently reported safety events. Fewer safety events were reported for IMI/REL across studies. These findings support the clinical use of IMI/REL and may inform Health Technology Assessment (HTA) decisions.

## 1. Background

Infections caused by Gram-negative bacteria, such as *Escherichia, Proteus, Enterobacterales,* and *Klebsiella* as part of the Enterobacteriaceae group, as well as *Pseudomonas aeruginosa* and *Acinetobacter baumannii* as part of the non-fermenters group, are a growing concern globally [1]. Gram-negative bacteria are a major driver of hospital-acquired/ventilator-associated bacterial pneumonia (HABP/VABP), complicated urinary tract infection (cUTI), and complicated intra-abdominal infection (cIAI) [2]. Treatment decisions for these infections are complicated by antimicrobial resistance (AMR), which occurs following bacterial exposure to an antimicrobe, creating selective pressure and resulting in mutations over time, which creates drug-resistant variants [3]. AMR arises when antibiotic exposure creates selective pressure that drives the emergence of drug-resistant variants via chromosomal mutation and by horizontal gene transfer (e.g., conjugation, transduction, transformation) [4]. AMR renders standard antimicrobials ineffective, prolongs infections, increases transmission risk, and leads to higher morbidity and mortality rates [5]; AMR was associated with an estimated 4.95 million deaths globally in 2019 [6]. The World Health Organisation’s Priority Pathogen List, updated in 2024, continues to rank multidrug-resistant Gram-negative infections as a critical priority for antimicrobial development [6,7,8,9,10]. Notably, *Pseudomonas aeruginosa* and *Acinetobacter baumannii* remain highly prevalent and problematic in this context [6,7,8,9,10,11].

Historically, polymyxins (Polymyxin A (colistin) and Polymyxin B) and aminoglycosides have been used as last-line agents to treat MDR Gram-negative infections, often in combination with other antibiotics such as anti-pseudomonal β-lactams [12]. This is because the clinical utility of polymyxins and aminoglycosides is constrained by well-documented toxicities, which range depending on factors such as dosage, underlying risk factors, and setting. Polymyxins have been shown to induce nephrotoxicity, with incidence varying by dose. For example, ≥300 mg/day colistin is associated with a significantly higher AKI risk, with high-dose colistin reporting nephrotoxicity rates of approximately 77.0%, versus reported nephrotoxicity rates for polymyxin B (approximately 25.0%) [13,14]. ICU rates have been reported as approximately 54.0–64.0% versus 36.0% in clinical trial populations, reflecting the compounding influence of critical illness severity, concomitant nephrotoxin exposure, and sepsis-related renal injury [13,14,15,16,17].

As well as nephrotoxicity, additional adverse events such as genitourinary complications further complicate the use of polymyxins and aminoglycosides, contributing to treatment discontinuations and resulting in these agents being positioned as later-line options, despite their broad activity against MDR infections [18,19,20,21]. However, the increase in AMR has led to the renewed use of polymyxins and aminoglycosides in clinical practice [12].

In response to these challenges, newer β-lactam/β-lactamase inhibitor combinations have been developed, such as imipenem/cilastatin/relebactam (IMI/REL), ceftazidime/avibactam, and meropenem/vaborbactam. IMI/REL was Food and Drug Administration (FDA)-approved for cUTI, cIAI, and HABP/VABP in July 2019 [20]. Contextualisation of efficacy and susceptibility is important to note to inform how the safety profile should be considered in settings where IMI/REL remains active against resistant Gram-negative pathogens. IMI/REL has demonstrated non-inferior efficacy versus colistin plus imipenem in the RESTORE-IMI 1 and 2 randomised trials, with differences observed in reported safety outcomes. IMI/REL also retains high in vitro activity against priority MDR pathogens and surveillance often shows >90% susceptibility for selected pathogens such as *Klebsiella pneumoniae carbapenemase* (KPC)-producing *Enterobacterales* and drug-resistant *Pseudomonas aeruginosa* [18,19,20,22,23].

## 2. Objectives

Given the significant safety concerns associated with polymyxins and aminoglycosides, as well as the emerging evidence supporting the safer profile of novel antibiotics such as IMI/REL, this targeted literature review (TLR) was conducted to synthesise current evidence on the adverse event profiles of these agents. The review captured any adverse events reported by publications, but reporting focused on nephrotoxicity, other toxicities (e.g., haematological, hepatological toxicities), renal adverse events, treatment-related mortality, and treatment-related discontinuation due to these being the most frequently reported adverse outcomes. This is with the goal of informing clinical decision-making and supporting better management of serious Gram-negative infections.

## 3. Materials and Methods

The TLR was conducted to identify and summarise available evidence relating to the safety profiles of aminoglycosides, polymyxins, and IMI/REL in the treatment of Gram-negative bacterial infections in adult patients. Eligibility criteria were predefined using a Population, Intervention, Comparator, Outcomes, and Study design (PICOS) framework to guide study identification and selection (Table 1). All included studies were assessed for risk of bias using Joanna Briggs Institute (JBI) critical appraisal tools appropriate to each study design. Owing to heterogeneity in study designs, populations, and outcome definitions, findings were summarised using descriptive synthesis without quantitative pooling. Detailed methods and search strategies are reported below, with risk-of-bias outputs presented in the Supplementary Information. In general, this TLR aligned with the principles informed by the Preferred Reporting Items for Systematic Reviews and Meta-Analyses (PRISMA) guidelines and the established methodological standards outlined in the Cochrane Handbook for Systematic Reviews of Interventions [24,25]. This TLR applied methodological elements consistent with PRISMA recommendations including prospectively defined inclusion and exclusion criteria, systematic search documentation, and senior review of screening outcomes, therefore enhancing methodological transparency and consistency [24].

### 3.1. Search Strategy

The TLR searches were run on 14 February 2025 and captured literature published between January 2015 and January 2025. The full search strategy is outlined in the Supplementary Material (Tables S1–S5). Across all databases, the search strategies were structured around a consistent set of core concepts. These included terms for Gram-negative bacterial infections, combined with antimicrobial resistance-related concepts. Safety concepts were incorporated using terms for adverse events, toxicity, and safety outcomes. Searches further restricted results to eligible clinical study designs, including randomised controlled trials and observational studies. Finally, treatment-specific terms were applied to capture the antibiotics of interest, including polymyxins (colistin and polymyxin B), aminoglycosides (e.g., gentamicin, amikacin, and tobramycin), and IMI/REL. The database search strategy combined free text searching and medical subject headings to ensure the most relevant literature was identified and reviewed. Literature searches were run via OVID^®^ and included Embase, MEDLINE^®^ (including MEDLINE Daily Update, In-Process and Other Non-Indexed Citations), EconLit, APA PsycINFO, and Evidence-Based Medicine (EBM) Reviews, covering DARE, ACP Journal Club, NHS EED, HTA, Cochrane Methodology Register, Clinical Answers, CENTRAL, and CDSR. The search was not restricted geographically, and targeted grey literature searches were also conducted (see Supplementary Materials for full search strategy).

The grey literature search identified publications from key conferences, including the European Congress of Clinical Microbiology and Infectious Diseases (ECCMID) and Infectious Disease (ID) Week, thereby capturing literature that has not yet been peer-reviewed. The search was conducted on 14 February 2025, and presented abstracts/posters from conferences that took place in the prior 3 years were reviewed. Key search terms, including those specific to treatments of interest, such as polymyxins, aminoglycosides, and IMI/REL, were used, along with search terms relating to key safety outcomes such as adverse events, mortality, and treatment discontinuation. Abstracts/posters presenting data already captured within articles included in the database search were excluded to prevent duplication.

### 3.2. Inclusion/Exclusion Criteria and Study Selection

The selection process was performed in two phases: title and abstract screening followed by full-text screening. The Population, Intervention, Comparator, Outcome, Time, and Study Design (PICOTS) criteria are shown in Table 1.

The PICOS criteria were specified as *a priori* in the study protocol to align with the clinical question of interest, which was to map the safety outcomes of agents used as alternatives for resistant Gram-negative infections. Interventions were restricted to polymyxins, aminoglycosides, and IMI/REL to reflect real-world decisions where these options are considered and to provide focus on the safety domains of highest clinical relevance (renal adverse events, toxicity, treatment-related discontinuation and mortality). Finally, this selection of interventions was included to maintain methodological consistency for descriptive synthesis due to heterogeneity in designs, dosing, and outcome definitions.

Safety outcomes were prespecified to focus on endpoints with standardised definitions and consistent reporting across study designs, namely renal adverse events (nephrotoxicity/AKI per RIFLE/AKIN/KDIGO), treatment-related discontinuation, and treatment-related mortality. These renal criteria are widely used and validated for cross study comparisons. Neurotoxicity was not included a priori due to the lack of a uniform outcome definition and inconsistent, often non-attributable reporting in the target populations, which would undermine comparability and risk of bias assessment.

Although the protocol considered paediatric populations, the executed review was restricted to adults (≥18 years) retrospectively because no relevant paediatric studies meeting the eligibility criteria were identified and available paediatric data were sparse and heterogeneous; this restriction preserves comparability of dosing, pharmacokinetics, and AKI risk across included studies.

The titles and abstracts of publications identified from the database and grey literature searches were screened by one researcher (AJ or JK) to determine eligibility according to the PICOTS criteria (Table 1). To ensure only the most relevant literature was reviewed, publications where it was not clear whether the pathogen reported on was a Gram-negative bacteria based on the title and abstract were excluded, as well as articles where it was not clear if safety events were reported for an intervention of interest based on the title and abstract. Following this, one researcher (AJ or JK) conducted a full-text review of publications to determine their eligibility according to the same inclusion and exclusion criteria as the title and abstract screening (Table 1). A random 20% sample was reviewed by a senior researcher (MS) to check for quality and consistency throughout the entire study selection process.

### 3.3. Data Collection, Analysis, and Assessment of Study Quality

The following categories of data were extracted into a bespoke data extraction form (DEF) developed in Microsoft Excel: study characteristics, population characteristics, and safety outcomes. Study characteristics extracted included study design, geographic location, healthcare setting, study period, sample size, follow-up duration, and study limitations. Population characteristics comprised patient demographics such as age and sex, baseline clinical characteristics, infection types and severity, pathogen identification methods, and resistance profiles. Further, intervention details were captured for interventions of interest, including dosing regimens, treatment duration, co-interventions, administration routes, and any comparator treatments.

Safety outcomes were categorised as primary and secondary endpoints. Primary outcomes extracted were the incidence and severity of renal adverse events (including nephrotoxicity, AKI, and renal replacement therapy), treatment discontinuation due to adverse events, and treatment-related mortality. Other toxicities, such as haematological adverse events (see Table 1), were categorised and extracted as secondary outcomes. These data extracted into the DEF informed the structure and conclusions presented in the reporting stage of the TLR.

All data were extracted by one reviewer (AJ or OM) and any uncertainty in the data was reviewed by a senior member of the research team (MS). A random 20% sample was reviewed by a senior researcher (MS) to check for quality and consistency. Due to heterogeneity in study designs, population characteristics, and outcome definitions, a formal meta-analysis was not conducted.

Although quantitative synthesis was considered, substantial clinical and methodological heterogeneity across studies, including differing designs, inconsistent nephrotoxicity definitions, and variable dosing and follow up, precluded meta-analysis. The review therefore presents a descriptive synthesis with study level results summarised narratively and in tables and figures. All publications underwent a quality assessment to determine the strength and applicability of the findings. Joanna Briggs Institute (JBI) critical appraisal tools appropriate to each study design were used to assess study quality and risk of bias. Quality assessments were documented and summarised to inform interpretation of findings [26]. Charts of appraisal, stratified by study type, are presented in Figures S1–S4 in the Supplementary Materials.

## 4. Results

### 4.1. Study Summary

**Figure 1:**
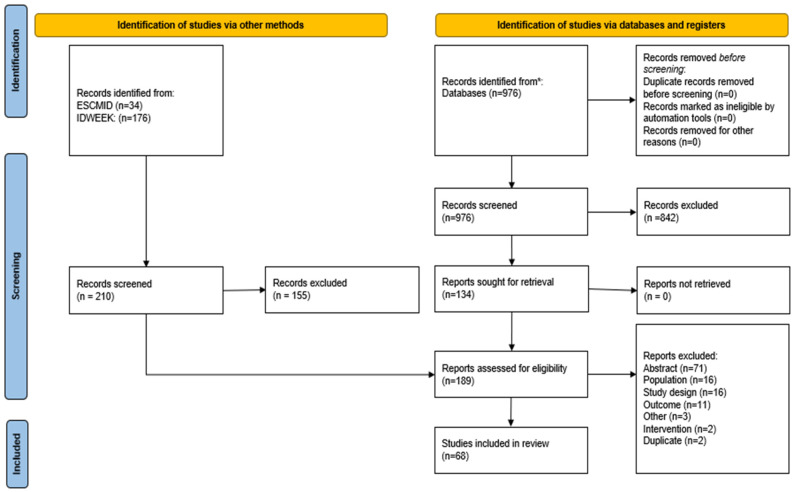
PRISMA figure of database searches and grey literature searches. * OVID^®^ and included Embase, MEDLINE^®^ (including MEDLINE Daily Update, In-Process and Other Non-Indexed Citations), EconLit, APA PsycINFO, and Evidence-Based Medicine (EBM) Reviews, covering DARE, ACP Journal Club, NHS EED, HTA, Cochrane Methodology Register, Clinical Answers, CENTRAL, and CDSR.

**Figure 2:**
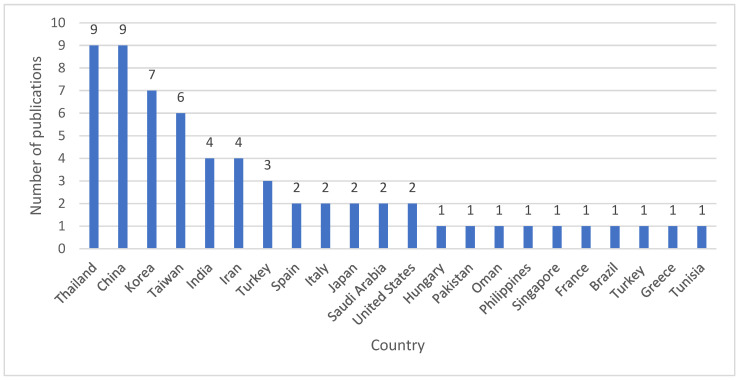
Geographical distribution of study settings for included publications (*n* = 68).

A total of 68 publications met the inclusion criteria for this TLR. An overview of the included publications based on the PICOTS criteria is presented in Figure 1. The majority were retrospective cohort studies (*n* = 48, 70.6%), supplemented by RCTs, (*n* = 14, 20.6%), prospective observational studies (*n* = 5, 7.4%), and one open-label observational study (*n* = 1, 1.5%). The publications were geographically diverse, with the highest representation from Thailand (*n* = 9, 13.2%), China (*n* = 9, 13.2%), and Korea (*n* = 7, 10.3%) (Figure 2).

PRISMA: Preferred Reporting Items for Systematic Reviews and Meta-Analyses.

### 4.2. Population Characteristics

Interventions included in publications were primarily colistin (*n* = 63 publications, 92.6%), Polymyxin B (*n* = 6 publications, 8.8%), and IMI/REL (*n* = 6 publications, 8.8%). Some publications reported multiple interventions of interest.

Publications primarily reported on populations with mixed infections (*n* = 13, 19.1%) or ventilator-associated pneumonia (VAP, *n* = 11 [16.2%]), although many did not specify the infection type (*n* = 23, 33.8%). Additional infection types included pneumonia (*n* = 7, 10.3%), hospital-acquired pneumonia (HAP, *n* = 4, 5.9%), respiratory tract infections (*n* = 2, 2.9%), and single publications each for bone/joint infections, UTI, pulmonary infections, bacteraemia, bloodstream infections, nosocomial pneumonia, cIAI, and cUTI. The 68 publications primarily reported carbapenem-resistant (*n* = 32, 47.0%), MDR (*n* = 14, 20.6%), and extensively drug-resistant (XDR) bacteria (*n* = 10, 14.7%). Some publications (*n* = 3, 4.4%) reported on mixed resistance types. Further, some publications did not report the resistance type (*n* = 9, 13.2%). A breakdown of the number of publications (*n* = 68) reporting on each pathogen type is presented in Table 2.

### 4.3. Safety Outcomes

A visual summary of the number of publications reporting on each safety outcome, stratified by intervention of interest, is presented in Table 3.

### 4.4. Renal Adverse Events

Nephrotoxicity was the most commonly reported endpoint across publications considered in this review, with 39 publications providing data for interventions of interest. The definition of nephrotoxicity varied between publications but commonly used Risk, Injury, Failure, Loss of Kidney Function, and End-stage (RIFLE) or Kidney Disease Improving Global Outcomes (KDIGO) criteria [66,71,72,75].

There were *n* = 37 publications reporting nephrotoxicity rates for intravenous colistin monotherapy, which ranged from 30.8% to 56.4%; higher rates were observed in elderly patients and critically ill populations. Combination therapies involving intravenous colistin sometimes showed even higher nephrotoxicity rates, with one publication, which reported on a total population sample size of 282 patients infected with CR-*Acinetobacter baumannii,* reporting a nephrotoxicity rate of 77.8% (*n* = 7/9) for IV colistin and amikacin in combination [44,71,75,79,86]. Further, high-dose colistin regimens (2.5–7 mg/kg or 300mg-9MIU daily) consistently demonstrated higher nephrotoxicity rates (ranging from 8.5% to 35.0%) compared to low-dose regimens (1.5–5 mg/kg or 200mg-2MIU daily; 6.1–10.0%) [37,41,42,45,61,77,78,81,87,88]. Aerosolised/inhaled colistin demonstrated inconsistent nephrotoxicity patterns compared with intravenous administration. For example, two publications reported lower nephrotoxicity rates with aerosolised delivery versus intravenous colistin (31.0% vs. 41.0% and 15.7% vs. 60.5%), while a third study, conducted in 191 patients infected with carbapenem-resistant Gram-negative bacteria with pneumonia in a hospital setting, found higher rates with aerosolised colistin (59.0%, *n* = 16/27) compared to intravenous regimens (38.0%, *n* = 23/61 (loading-dose colistin) to 54.0%, *n* = 27/50 (non-loading-dose colistin)) [29,37,59,89].

Among the included studies, four publications reported nephrotoxicity outcomes for Polymyxin B, and rates ranged from 14.6% to 54.9% [38,66,68,76]. Further, the nephrotoxicity rate for Polymyxin B occasionally exceeded colistin rates in comparative publications; however, there was no clear difference between the two interventions [38,66]. Regarding the evaluation of IMI/REL, one Phase III RCT (RESTORE-IMI 1) reported a nephrotoxicity incidence of 10.3% (*n* = 3/29), which was significantly lower within this individual trial than colistin plus imipenem (56.3%, *n* = 9/16 *p* = 0.002) [72].

Overall, 32 publications reported on renal impairment for the interventions of interest; this was defined inconsistently across publications, with some studies using renal risk/injury/failure criteria and others using AKD/AKI definitions. Renal impairment rates ranged from 15.0% to 53.8% in colistin-treated patients (*n* = 29 publications). Three publications reported Polymyxin B-associated renal impairment rates (which also included reporting the incidence of AKI). The reported renal impairment rates for this intervention ranged from 5.3% to 58.1% [52,67,76]. One comparative study, which reported on patients infected with CRE in an ICU setting, reported lower renal impairment rates with Polymyxin B versus colistin (5.3%, *n* = 1/19 vs. 10.0%, *n* = 2/20) [52].

The RESTORE-IMI 1 trial provided comprehensive renal impairment data using both the KDIGO and RIFLE criteria, demonstrating lower-reported renal impairment rates with IMI/REL compared to colistin plus imipenem [18,72]. Using KDIGO criteria, patients treated with IMI/REL (*n* = 29) experienced stage 1 AKI in 17.2% of cases, stage 2 AKI in 3.4% of cases, and stage 3 AKI in 0%, whereas colistin plus imipenem-treated patients (*n* = 16) experienced stage 1, 2, and 3 AKI in 37.5%, 12.5%, and 31.3% of cases, respectively. When RIFLE criteria were applied, 10.3%, 6.9%, and 0.0% of patients (*n* = 29) treated with IMI/REL experienced renal risk, injury, and failure compared to 37.5%, 12.5%, and 25.0% of patients (*n* = 16) treated with colistin, respectively [18,72].

Overall, eight publications reported on renal replacement therapy (RRT), with six reporting on colistin and two on Polymyxin B [37,40,49,67,68,77,81,86]. Polymyxin B showed consistently high RRT rates across two publications (31.3% to 32.6%), while colistin demonstrated more variable rates (2.6% to 43.8%) depending on multiple clinical factors including treatment response and dosing regimen [31,44,49,67,68,72,76].

### 4.5. Discontinuation and Mortality

Overall, five publications reported treatment discontinuation, explicitly related to the antimicrobial treatment or its complications, as a safety outcome of interest [20,50,64,69,80,85]. Colistin regimens demonstrated discontinuation rates ranging from 12.5% to 23.0%, often driven by nephrotoxicity and other toxicities [20,26]. IMI/REL showed minimal discontinuation rates (0.0% to 2.3%) due to adverse events [20,64,69]. Treatment discontinuation was not reported for Polymyxin B across included publications.

Overall, treatment-related mortality, defined as mortality explicitly related to the antimicrobial treatment or its complications, was specified in six publications. Publications reporting on colistin monotherapy reported mortality rates ranging from 32.0% to 39.1%, as identified in ICU and hospital patients with VAP caused by *Acinetobacter baumannii* [40,44,79]. Colistin combination therapy was also associated with high mortality with colistin plus ampicillin/sulbactam demonstrating a treatment-related mortality rate of 25.0% (N = 4/20) in one publication reporting on outcomes for patients with CRE who experienced mortality due to deteriorating clinical findings of VAP or septic shock in an ICU setting [40]. Further, treatment-related mortality ranged from 0.0% to 0.7% in patients treated with IMI/REL [20,64,69].

### 4.6. Hospitalisation and Critical Care Admissions

Per the protocol, economic and healthcare resource utilisation (HCRU) safety outcomes were captured when reported; however, these data were limited and heterogeneous across included studies. Overall, three publications reported on hospital/ICU LOS explicitly associated with treatments of interest and complications [33,78,90].

Across the reviewed literature, hospital and ICU admissions and LOS varied depending on the combination, dosage, and formulation of colistin used and ranged from 12 days to 56 days [33,78,90]. In a retrospective study conducted in a population of 320 patients in a hospital care setting, colistin monotherapy was associated with a median hospital LOS of 12 days, whereas colistin-based combination therapy was linked to longer hospital LOS ranging from 18 to 22 days, depending on the regimen (*p* < 0.001) [33]. In a publication reporting on 127 patients with CR Gram-negative infection, colistin dose variation (low versus high dosage) did not significantly affect hospital LOS, with both regimens showing a median LOS of 16 days [78]. In a publication reporting on 195 patients infected with CRE in a critical care setting, colistin formulations impacted outcomes; patients treated with Locolin^®^ had a median ICU stay of 30 days (IQR: 19–47), while those who developed AKI following treatment experienced extended ICU stays up to 60 days (IQR: 38–94 days) [90]. In the same publication, the median ICU LOS for patients treated with Colimycin^®^ was 31 days (IQR: 21–54 days) [90]. Median hospital LOS was 56 days (IQR: 35–87) for Locolin^®^ and 50 days (IQR: 29–76) for Colimycin^®^ [90].

### 4.7. Other Toxicities

Haematological toxicities were reported in four publications, three of which evaluated colistin-based regimens, with one of these reporting on colistin versus Polymyxin B; the fourth publication reported on IMI/REL [41,60,64,66]. In a retrospective study conducted in patients with MDR Gram-negative bacteria, colistin resulted in lower rates of platelet decline (13.3%, *n* = 4/30) than Polymyxin B (21.2%, *n* = 10/47) [66]. One multinational Phase III randomised controlled trial (RESTORE-IMI 2) reported that 1.5% (*n* = 4/266) of patients experienced thrombocytopenia with IMI/REL [64].

Hepatological toxicities were reported on by three publications, one of which evaluated colistin-based regimens versus Polymyxin B and two of which reported on IMI/REL [20,64,66]. In one retrospective study conducted in patients with MDR Gram-negative bacteria in an ICU setting, the rate of increased aspartate aminotransferase was 28.1% for patients treated with colistin (*n* = 9/32), compared to 10.9% for patients treated with Polymyxin B (*n* = 5/46) [66]. In the same publication, the rate of increased alanine aminotransferase was 13.3% for patients treated with colistin (*n* = 4/30) compared to 10.0% for patients treated with Polymyxin B (*n* = 5/50) [66]. In one publication reporting on a multinational Phase III randomised controlled trial (RESTORE-IMI 1), increased aspartate aminotransferase was reported for 9.7% of patients (*n* = 3/31) and increased alanine aminotransferase was reported for 6.5% of patients (*n* = 2/31) treated with IMI/REL [20]. In the same trial, both increased aspartate aminotransferase and increased alanine aminotransferase were reported for 18.8% of patients (*n* = 3/16) for patients treated with colistin + IMI/REL [20]. In a final publication reporting on a multinational Phase III randomised controlled trial (RESTORE-IMI 2), abnormal hepatic function was reported for 0.4% of patients (*n* = 1/266) treated with IMI/REL [64]. Increased aspartate aminotransferase and increased alanine aminotransferase were both reported for 2.3% of patients (*n* = 6/266) for patients treated with IMI/REL in the same trial [64].

Leukopenia was reported in two studies, with outcomes reported for colistin and colistin + IMI/REL [20,60]. In one study reporting on 61 patients infected with CRE in a hospital setting, 37.9% (*n* = 11/29) of patients treated with colistin experienced white blood cell normalisation compared to 57.4% (*n* = 19/32) of patients treated with ceftazidime-avibactam (*p* = 0.09) [60]. In the RESTORE-IMI-1 trial, which was a multicentre, randomised, double blind trial reporting on a total population sample size of 47 patients in a hospital care setting, 0.0% (*n* = 0/31) of patients treated with IMI/REL experienced leukopenia versus 6.3% (*n* = 1/16) of patients treated with colistin+ IMI/REL [20].

One publication reported on urinary tract disorders (UTI). In this publication reporting on a total sample population of 207 patients with CRE in a hospital setting, UTI rates were reported as 31.0% (*n* = 27/85) for patients treated with intravenous colistin [28].

*Clostridioides difficile* infection (CDi) was reported on by four publications [12,64,72,82]. In one publication reporting on 51 patients with XDR infection in an ICU setting, rates of CDi ranged from 0.0% (for a combination colistin + aminoglycoside therapy) to 9.1% (*n* = 3/33) [82]. For one publication on the RESTORE-IMI 2 trial evaluating outcomes for IMI/REL, 0.4% of patients (*n* = 1/266) were reported to have clostridium difficile colitis [64].

Eligible studies rarely reported neurotoxicity with explicit treatment attribution, and therefore it was not synthesised in this review.

Ototoxicity data were sparse and inconclusive.

### 4.8. Comparison of Different Populations by Type of Regimen

In ten publications reporting safety outcomes for MDR populations, the incidence of nephrotoxicity ranged from 10.0% to 56.4% and the incidence of renal impairment ranged from 0.0% to 58.0% [45,59,61,65,66,71,74,75,77,81]. In six publications reporting safety outcomes in XDR populations, the incidence of nephrotoxicity ranged from 18.5% to 43.5% and renal impairment incidence ranged from 35.6% to 35.9% [12,29,44,82,83,86]. In 20 publications reporting on CRE populations, nephrotoxicity rates ranged from 4.9% to 64.3% while rates of renal impairment ranged from 9.1% to 53.8% [28,30,31,32,47,48,49,50,51,52,53,56,57,60,62,68,78,87,88,90].

Across populations with MDR, XDR, or CRE Gram-negative infections, colistin consistently demonstrated high nephrotoxicity rates, which ranged from 10.0% to 64.3%, and renal impairment rates ranging from 9.1% to 58.0%. Further, Polymyxin B demonstrated nephrotoxicity rates of 14.6% to 15.6% and renal impairment rates of 5.3% to 15.6% in patients with MDR, XDR, or CRE Gram-negative infections [52,66,68]. Publications reporting on IMI/REL reported on “non-susceptible infections” but did not provide a breakdown on resistance profiles [20,23,32,64,69,85].

### 4.9. Risk of Bias

Risk of bias was assessed using the JBI critical appraisal tools for case control studies (*n* = 1), cohort studies (*n* = 48), RCTs (*n* = 14), and prevalence studies (*n* = 5). For case control studies, the included study addressed risk of bias across nine categories. For the five prevalence studies that were included, *n* = 5 (100.0%) of the studies addressed all nine categories. Overall, the included studies that were cohort and RCT studies demonstrated strong methodological quality across categories. All RCTs employed true randomisation and intention-to-treat analysis, while ≥97.0% of cohort studies met criteria for valid exposure and outcome measurement, adequate follow-up and group comparability. However, notable limitations were observed. Among cohort studies, a minority reported strategies to address confounding factors (*n* = 8, 16.7%) despite most identifying relevant confounders (*n* = 43, 89.6%), and few used methods to handle incomplete follow-up (*n* = 4, 8.3%). Among RCTs, blinding was consistently underreported, with fewer than half reporting adequate blinding (6, 42.9%) and fewer than one-third achieving participant (5, 35.7%), treatment deliverer (4, 28.6%), or outcome assessor blinding (4, 28.6%).

## 5. Discussion

This TLR identified 68 publications summarising evidence relating to the safety burden associated with polymyxins, aminoglycosides, and IMI/REL in the treatment of Gram-negative infections. Of note, the most frequently reported intervention of interest was colistin (*n* = 63 publications). This may be due to the long-term establishment of colistin as the standard of care for the treatment of Gram-negative infections [12]. Over recent years, there have been increasing safety and resistance concerns with the use of polymyxins and aminoglycosides, which remain mainstays of treatment for several critical indications, such as HABP/VABP, cUTI, and cIAI [91]. However, more recently developed IMI/REL is associated with lower reported rates of adverse events and safety outcomes, relative to aminoglycoside- or polymyxin-based combinations [92,93,94,95]. It is therefore imperative to understand the broader burden associated with these therapies to ensure clinicians understand the full impact of their use.

Renal adverse events were the most common safety outcomes identified within the TLR, including nephrotoxicity (reported on by *n* = 39 publications), renal impairment (reported on by *n* = 32 publications), and use of RRT (reported on by *n* = 8 publications). This TLR identified high incidence of nephrotoxicity (range: 30.0% to over 50.0%) and renal impairment (range: 15.0% to over 50.0%) with use of IV colistin. However, the reported impact of inhaled or aerosolised colistin on renal function was inconsistent. Within the literature, evidence suggests that IV administration of colistin is more likely to result in AEs such as nephrotoxicity compared to inhaled and aerosolised administration routes, although there are differences in the types of AEs associated with colistin across different administration routes [96]. Further, this TLR identified that nephrotoxicity rates depend on the dose of colistin, with high-dose colistin consistently producing higher renal impairment rates than low-dose regimens [37,41,42,45,61,77,78,81,87,88]. Notably, combination therapies involving colistin plus aminoglycosides demonstrated high nephrotoxicity rates, with the combination of colistin plus amikacin reaching 77.8% [34]. Similarly, of the nine publications included in the TLR that reported on Polymyxin B, four publications reported renal AEs with the use of Polymyxin B and identified a similar or higher incidence of nephrotoxicity compared with colistin [38,66]. These findings indicate that while colistin, Polymyxin B, and aminoglycosides remain options for Gram-negative infections, their nephrotoxic potential warrants consideration of alternative or adjunctive agents to minimise renal AEs. Thus, their safety implications constrain their clinical usability [97]. Further, the literature for aminoglycosides was limited but also suggested low tolerability.

IMI/REL is a newer agent approved by the FDA for the treatment of adult patients with cUTIs, cIAIs, and HABP/VABP [98,99,100,101]. The TLR identified an RCT (RESTORE-IMI 1) conducted in patients with Gram-negative infections that compared IMI/REL (*n* = 16) with colistin plus IMI (*n* = 29) across 16 countries [20]. The RCT reported that treatment-emergent nephrotoxicity was significantly less frequent (*p* = 0.002) with IMI/REL (10.3%) than with colistin + IMI (56.0%), respectively (95% CI for difference, –69.1%, –18.4%) [72]. This highlights the potential safety advantages, in terms of renal toxicity, of using IMI/REL versus colistin and Polymyxin B for the treatment of GN infections.

Treatment-related mortality varied across interventions. Polymyxin-based regimens demonstrated considerable mortality risks; an open-label prospective study reported that colistin monotherapy was associated with 32% mortality in patients with VABP versus 25.0% for colistin plus ampicillin/sulbactam in combination [40]. Additionally, the highest reported mortality rate in this review was 39.1%, in a retrospective observational study conducted for a total population sample size of 329 patients with XDR Gram-negative bacteria, infected with *Acinetobacter baumannii* in a hospital setting. The highest reported mortality rate of 39.1% occurred in the nephrotoxicity subgroup (*n* = 56/143), for patients treated with intravenous colistin [44]. However, IMI/REL demonstrated a treatment-related mortality of 0.0% to 0.7% across two adequately powered (*n* > 100) multinational Phase III trials (*n* = 134–266) in patients with HAP/VAP treated in a hospital setting, supporting the intervention’s clinical positioning as a potentially safer therapeutic alternative for serious Gram-negative infections [20,72].

Polymyxins and aminoglycosides were consistently associated with high rates of nephrotoxicity and mortality across MDR, XDR, and CRE populations, complicating their use in treatment of Gram-negative infections. Alternatively, IMI/REL maintained lower reported rates of adverse safety outcomes across the included publications for resistant Gram-negative infections; a systematic review and meta-analysis of 282,621 isolates found that the prevalence rate of IMI/REL resistance is roughly 14.6%, indicating that resistance to IMI/REL is infrequent [102]. As a result, the WHO Expert Committee supported the inclusion of IMI/REL as part of the Reserve Medicines list due to its coverage across MDR and carbapenem-resistance profiles [103]. As AMR is increasingly a globally concern, this further supports the evidence basis for the clinical use of IMI/REL for (resistant) Gram-negative infections. Head-to-head RCTs comparing IMI/REL with optimised polymyxin/aminoglycoside dosing strategies are needed to validate the tolerability of IMI/REL versus older agents in a range of infection types and clinical settings.

This review planned to evaluate the feasibility of quantitative synthesis (including Bayesian methods via R/WinBUGS) per the protocol; however, key sources of heterogeneity, inconsistent AKI/nephrotoxicity definitions (RIFLE/AKIN/KDIGO/author-defined), varied monitoring and attribution methods, a mix of RCTs and observational cohorts with differing confounding risk, and sparse reporting of some outcomes (e.g., LOS), precluded quantitative pooling. Consequently, this review presented a structured narrative synthesis and study-level data tables to enable transparent interpretation while avoiding potentially misleading pooled estimates. Additionally, per the protocol, this review attempted to capture economic and HCRU outcomes, but the heterogeneous nature of reported data precluded quantitative synthesis or robust comparative analysis. Therefore, economic and HCRU findings were sparse.

## 6. Limitations

There were some limitations of this review. Firstly, this review was a TLR, in which the methodology warranted less comprehensive review of the evidence, compared to a systematic literature review (SLR). As this was a targeted literature review, single reviewer screening with 20% validation may introduce a degree of study selection bias. However, this risk was mitigated by senior reviewer oversight and consistency checks. Additionally, the search strategy and screening process were designed and executed to identify the most relevant literature and therefore, this TLR may not have identified all applicable literature available.

Secondly, as this was a targeted review using a priori PICOS focused on three intervention categories, findings could not be generalised to other novel agents that were not included in the review.

Neurotoxicity was not prespecified because it lacked consistent definitions across the included studies, and the evaluation of this outcome is complicated in critical-care settings where symptoms may overlap, for example, with sepsis or metabolic disturbances. As neurotoxicity was inconsistently defined and reported, available findings could not be synthesised using the same structured approach applied to other safety outcomes.

In addition, substantial clinical and methodological heterogeneity was observed across the included studies, including differences in study design, healthcare settings, patient populations, antimicrobial treatment regimens, and assessment methods for renal safety outcomes (e.g., KDIGO, RIFLE, or investigator-defined nephrotoxicity). This heterogeneity precluded quantitative pooling of results and limits the direct comparability of safety outcomes across studies.

Several of the studies included were non-randomised observational studies, which are subject to confounding. Differences in baseline illness severity, infection type, renal function, concomitant nephrotoxic medications, and dosing or combination therapy may have influenced observed safety outcomes and could not be consistently accounted for across studies.

Finally, only six publications reported on IMI/REL, and two publications reported on safety outcomes for aminoglycosides. For publications which did report on IMI/REL, patient numbers were relatively small (<100). In relation to this, as colistin was evaluated in the majority of included publications (*n* = 63) compared with a much smaller evidence base for IMI/REL (*n* = 6), the level of detail presented in the results reflects the distribution of available studies rather than emphasis on any specific intervention. Further, significant heterogeneity existed across publications in patient populations, infection types/pathogens and resistance profiles, limiting indirect treatment comparison. The follow-up periods and safety outcomes/definitions also demonstrated significant heterogeneity across identified literature.

## 7. Conclusions

This TLR, which includes 68 publications, demonstrates that polymyxins are frequently associated with a substantial reported safety burden in the treatment of Gram-negative infections, including renal adverse events, treatment-related mortality, treatment discontinuation, and other toxicities. Publications reporting on IMI/REL noted numerically fewer treatment-related safety events. As antimicrobial resistance increases globally, these findings may support clinical decision making by outlining the safety profiles reported in the available evidence and highlight IMI/REL as an important therapeutic option for resistant Gram-negative infections.

## Figures and Tables

**Table 1:** PICOTS criteria for inclusion/exclusion of publications for the TLR.

	Inclusion Criteria	Exclusion Criteria
Population(s)	Adult patients receiving treatment for GN bacterial infections.	Populations without GN bacterial infections or where the pathogen group is not stated Paediatric populations
Interventions	Polymyxins: ○ Colistin (Polymyxin E) ○ Poly-Rx (Polymyxin B) Aminoglycosides: ○ Gentamicin ○ Amikacin ○ Tobramycin Imipenem/cilastatin/relebactam (IMI/REL)	Any interventions not listed in the inclusion criteria
Comparators	Any	NA
Outcomes	Incidence and severity of any adverse event, particularly: ○ Nephrotoxicity ^1^ ○ Renal impairment (including AKI) ^1^ ○ Ototoxicity ○ Leukopenia ○ Genitourinary ○ Treatment-related discontinuation ^2^ ○ Treatment-related mortality ^2^ ○ Including: economic, clinical and healthcare resource use (e.g., treatment-related length of stay) ^3^	Any outcome not in the inclusion criteria (e.g., PK/PD outcomes, susceptibility outcomes) Mortality outcomes that are not explicitly related to the treatment (e.g., all-cause mortality, in-hospital mortality, 30-day mortality, with no explicit link to treatment)
Time	Studies published from January 2015 to January 2025	Studies published prior to January 2015
Study Design	Randomised controlled trials (RCTs) Non-randomised controlled clinical studies (e.g., non-interventional observational studies, cohort studies, case control studies) ^4^ Economic evaluations (e.g., cost-effectiveness/BIM), where primary data is used to model the outcomes	Case studies and studies including a patient population size of less than 20 sample size Non-clinical studies (e.g., in vitro) Commentaries, editorials, literature reviews, systematic literature reviews, meta-analysis, opinion articles, anecdotal reports, or letters without data presented Abstracts and letters to the editor PK/PD studies Economic evaluations which utilise secondary data
Other	English language publications, human studies	Studies not published in English, non-human studies

AKI: Acute kidney injury; BIM: Budget impact model; N/A: Not applicable; PD: Pharmacodynamics; PK: Pharmacokinetics; RCT: Randomised controlled trial; SAE: Serious adverse event. ^1^ Definitions of nephrotoxicity varied across publications. Typically, KDGIO and RIFLE or investigator-reported nephrotoxicity were utilised. Renal impairment was defined as renal injury/risk/failure or incidence of acute kidney injury (AKI). ^2^ Treatment-related mortality and treatment-related discontinuation are defined as deaths or discontinuation attributed by study investigators as explicitly related to the antimicrobial treatment or its complications. ^3^ Length of stay (hospital and Intensive Care Unit (ICU) LOS) attributed by study investigators as explicitly related to the antimicrobial treatment or its complications; data were limited (*n* = 3) and heterogeneous across studies. ^4^ A non-randomised controlled study: A non-randomised controlled study is a type of study where participants are not randomly assigned to different groups or interventions. Instead, participants are assigned based on factors such researchers’ discretion, patient preference, or availability. Non-randomised controlled studies can include observational studies, cohort studies, case control studies, and quasi-experimental studies. These studies aim to compare the outcomes of different groups or interventions but lack the random assignment of participants.

**Table 2:** Number of publications (*n* = 68) reporting on each pathogen type as the primary pathogen.

Pathogens	Number of Publications
*A. baumannii*	26 [27,28,29,30,31,32,33,34,35,36,37,38,39,40,41,42,43,44,45,46,47]
Mixed	21 [18,48,49,50,51,52,53,54,55,56,57,58,59,60,61,62,63,64,65,66,67]
NR	15 [68,69,70,71,72,73,74,75,76,77,78,79,80,81]
*P. aeruginosa*	4 [82,83,84]
*K. pneumoniae*	1 [48]
*E. coli*	1 [85]

*A. baumannii*: Acinetobacter baumannii. *P. aeruginosa*: Pseudomonas aeruginosa. *E. coli*: *Escherichia coli. K. pneumoniae*; *Klebsiella pneumoniae.* NR: Not reported; Mixed: Publications which reported on multiple pathogens.

**Table 3:** Number of publications reporting on safety outcomes, stratified by intervention (N = 68).

Safety Outcome	Drug	Number of Publications
Nephrotoxicity	Colistin	37
Nephrotoxicity	Polymyxin B	2
Nephrotoxicity	IMI/REL	2
Renal impairment	Colistin	29
Renal impairment	Polymyxin B	3
Renal Replacement Therapy	Colistin	6
Renal Replacement Therapy	Polymyxin B	2
Treatment discontinuation	Colistin	1
Treatment discontinuation	IMI/REL	2
Treatment-related mortality	Colistin	3
Treatment-related mortality	Polymyxin B	1
Treatment-related mortality	IMI/REL	2
Hematological Toxicity	Colistin	3
Hematological Toxicity	Polymyxin B *	1
Hematological Toxicity	IMI/REL	1
Hepatological Toxicity	Colistin	1
Hepatological Toxicity	Polymyxin B	1
Hepatological Toxicity	IMI/REL	2
Leukopenia	Colistin	1
Leukopenia	IMI/REL	1
C diff	Colistin	3
C diff	IMI/REL	1
C diff	Amikacin **	1
UTI	Colistin	1

* Refers to one publication (Wang et al., 2023) which reported on haematological and hepatological outcomes for colistin and Polymyxin B separately in the same publication [66]. ** Refers to one publication which reported on C. diff outcomes for amikacin and IV colistin separately in the same publication [12]. C. diff: *Clostridium difficile*; IMI/REL: Imipenem/cilastatin/relebactum; UTI: Urinary tract infection.

## Data Availability

No new data were created or analysed in this study. Data sharing is not applicable to this article.

## Supplementary Materials

The following supporting information can be downloaded at https://www.mdpi.com/article/10.3390/antibiotics15050422/s1.

## Abbreviations

AEs: Adverse events; AKI: Acute kidney injury; ALT: Alanine aminotransferase; AMR: Antimicrobial resistance; AST: Aspartate aminotransferase; BIM: Budget impact model; CDi: *Clostridioides difficile* infection; Cr: Creatinine; CRE: *Carbapenem*-resistant *Enterobacterales*; CR GNB: *Carbapenem*-resistant Gram-negative bacteria; DEF: Data extraction form; EBM: Evidence-based medicine; FDA: Food and Drug Administration; GFR: Glomerular filtration rate; GN: Gram-negative; HABP: Hospital-acquired bacterial pneumonia; HAP: Hospital-acquired pneumonia; HCRU: Healthcare resource use; ICU: Intensive Care Unit; IMI/REL: Imipenem/cilastatin/relebactam; JBI: Joanna Briggs Institute; KDIGO: Kidney Disease: Improving Global Outcomes; KPC: *Klebsiella pneumoniae carbapenemase*; LOS: Length of stay; MDR: Multidrug-resistant; NR: Not reported; OR: Odds ratio; PICOS/PICOTS: Population, Intervention, Comparator, Outcomes, Time, Study design; PK: Pharmacokinetics; RCT: Randomised controlled trial; RIFLE: Risk, Injury, Failure, Loss, End stage kidney disease; RRT: Renal replacement therapy; SAE: Serious adverse event; SLR: Systematic literature review; TLR: Targeted literature review; UTI: Urinary tract infection; VABP: Ventilator-associated bacterial pneumonia; VAP: Ventilator-associated pneumonia; XDR: Extensively drug-resistant.
