# Supplementary material for: Safety Profiles of Polymyxins, Aminoglycosides, and Imipenem/Cilastatin/Relebactam (IMI/REL) in the Treatment of Gram-Negative Infections: A Literature Review
Source: Antibiotics (Basel). 2026 Apr 22;15(5):422. doi: 10.3390/antibiotics15050422 (PMC13203158; doi:10.3390/antibiotics15050422)
Supplement: Supplementary file 1 [file antibiotics-15-00422-s001.zip › antibiotics-4109437-supplementary.pdf]

Supplementary information

Table S1. Search strategy: Embase

#	Query	Results from 18 Feb 2025
1	exp Gram-Negative Bacterial Infections/ or exp Gram negative infection/	370,452
2	(Gram negative or Gram-negative or GN).ti,ab.	139,868
3	Antibiotic resistance/ or Drug resistance, bacterial/ or beta-lactam resistance/ or Drug resistance, multiple, bacterial/ or carbapenem-resistant Enterobacteriaceae/	262,327
4	(((carbapenem or meropenem or imipenem) adj2 resistan*) or beta-lactamase or carbapenemase or ESBL or ((multi-drug or multi drug) adj2 resistan*) or ((pan-drug or pan drug) adj2 resistan*) or ((extensive* drug or extensive-drug or extensively-drug) adj2 resistan*)).ti,ab.	82,882
5	1 or 2	494,690
6	3 or 4	304,894
7	5 and 6	57,025
8	exp adverse event/	1,137,265
9	exp safety/	1,261,498
10	(adverse reaction or safety profile or side effect).mp. [mp=title, abstract, heading word, drug trade name, original title, device manufacturer, drug manufacturer, device trade name, keyword heading word, floating subheading word, candidate term word]	1,385,169
11	exp toxicity/	836,918
12	8 or 9 or 10 or 11	3,452,797
13	exp Clinical Trial/ or exp Randomised Controlled Trial/ or exp controlled clinical trial/ or exp multicenter study/ or exp Phase 3 clinical trial/ or exp Phase 4 clinical trial/ or exp Phase 2 clinical trial/ or exp randomization/ or exp Single Blind Procedure/ or exp Double Blind Procedure/ or exp Crossover Procedure/ or randomi?ed controlled trial$.mp. or rct.mp. or (random$ adj2 allocat$).mp. or single blind$.mp. or double blind$.mp. or ((treble or triple) adj blind$).mp. or exp Prospective Study/	3,217,249
14	case control study/ or observational study/ or cross-sectional study/ or health survey/ or longitudinal study/ or cohort analysis/ or meta analysis/	6,969,944

	or (prospective adj (study or studies)).mp. or (retrospective adj (study or studies)).mp. or (longitudinal adj (study or studies)).mp. or (cohort adj (study or studies)).mp. or (case control adj (study or studies)).mp. or (follow up adj (study or studies)).mp. or (epidemiologic* adj (study or studies)).mp. or (cross sectional adj (study or studies)).mp. or (survey* or surveillance).mp. or (meta adj analys#s).mp.	
15	13 or 14	8,656,295
16	(polymixinADJ1B or polymixinADJ1 E or colistin or Gentamycine or Amikacine or Tobramycine or streptomycin or ((imipenem adj1 cilastatin adj1 relebactam) or RECARBRIO or MK-7655A)).mp.	92,559
17	7 and 16	9,484
18	7 and 12 and 15 and 16	963

Table S2. Search strategy- MEDLINE

#	**Query**	**Results from 18 Feb 2025**
1.	exp Gram-Negative Bacterial Infections/ or exp Gram-Negative Bacteria/	1,064,334
2.	(Gram negative or Gram-negative or GN).ti,ab.	113,524
3.	Drug resistance, microbial/ or Drug resistance, bacterial/ or beta-lactam resistance/ or Drug resistance, multiple, bacterial/ or carbapenem-resistant Enterobacteriaceae/	145,733
4.	(((carbapenem or meropenem or imipenem) adj2 resistan*) or beta-lactamase or carbapenemase or ESBL or ((multi-drug or multi drug) adj2 resistan*) or ((pan-drug or pan drug) adj2 resistan*) or ((extensive* drug or extensive-drug or extensively-drug) adj2 resistan*)).ti,ab.	64,342
5.	1 or 2	1,119,642
6.	3 or 4	186,381
7.	5 and 6	90,964
8.	exp safety/	91,369
9.	(adverse reaction or safety profile or side effect).mp. [mp=title, book title, abstract, original title, name of substance word, subject heading word, floating sub-heading word, keyword heading word, organism supplementary concept word, protocol supplementary concept word, rare disease supplementary concept word, unique identifier, synonyms, population supplementary concept word, anatomy supplementary concept word]	99,058
10.	8 or 9	189,308

11.	exp Clinical Trial/ or exp Randomised Controlled Trial/ or exp controlled clinical trial/ or multicenter study/ or clinical trial, phase III/ or clinical trial, phase IV/ or clinical trial, phase II/ or random allocation/ or exp Single-Blind Method/ or Double-Blind Method/ or exp Cross-over studies/ or randomi?ed controlled trial$.mp. or rct.mp. or (random$ adj2 allocat$).mp. or single blind$.mp. or double blind$.mp. or ((treble or triple) adj blind$).mp. or exp Prospective Study/	2,183,690
12.	Case control studies/ or observational study/ or cross-sectional studies/ or health surveys/ or longitudinal studies/ or cohort studies/ or meta-analysis/ or (prospective adj (study or studies)).mp. or (retrospective adj (study or studies)).mp. or (longitudinal adj (study or studies)).mp. or (cohort adj (study or studies)).mp. or (case control adj (study or studies)).mp. or (follow up adj (study or studies)).mp. or (epidemiologic* adj (study or studies)).mp. or (cross sectional adj (study or studies)).mp. or (survey* or surveillance).mp. or (meta adj analys#s).mp.	5,371,117
13.	11 or 12	6,409,733
14.	(polymixinADJ1B or polymixinADJ1 E or colistin or Gentamycine or Amikacine or Tobramycine or streptomycin or ((imipenem adj1 cilastatin adj1 relebactam) or RECARBRIO or MK-7655A)).mp.	45,607
15.	7 and 14	9,596
16.	7 and 10 and 13 and 14	19

Table S3. Search strategy- Econlit

#	Query	Results from 18 Feb 2025
1.	(Gram negative or Gram-negative or GN).ti,ab.	18

Table S4. Search strategy- PsycInfo

#	Query	Results from 19 Feb 2025
1.	exp Bacterial Infections/	3,455
2.	(Gram negative or Gram-negative or GN).ti,ab.	408
3.	Drug resistance/	169

4.	(((carbapenem or meropenem or imipenem) adj2 resistan*) or beta-lactamase or carbapenemase or ESBL or ((multi-drug or multi drug) adj2 resistan*) or ((pan-drug or pan drug) adj2 resistan*) or ((extensive* drug or extensive-drug or extensively-drug) adj2 resistan*)).ti,ab.	150
5.	1 or 2	3,832
6.	3 or 4	311
7.	5 and 6	56
8.	exp "side effects (drug)"/	82,412
9.	exp safety/	68,971
10.	(adverse reaction or safety profile or side effect).mp. [mp=title, abstract, heading word, table of contents, key concepts, original title, tests & measures, mesh word]	9,020
11.	exp toxicity/	8,271
12.	8 or 9 or 10 or 11	154,027
13.	exp Clinical Trials/ or exp Randomised Controlled Trials/ or random sampling/ or randomi?ed controlled trial$.mp. or rct.mp. or (random$ adj2 allocat$).mp. or single blind$.mp. or double blind$.mp. or ((treble or triple) adj blind$).mp. or Prospective Studies/	111,690
14.	Case control studies/ or observational study/ or cross-sectional studies/ or surveys/ or longitudinal studies/ or meta analysis/ or (prospective adj (study or studies)).mp. or (retrospective adj (study or studies)).mp. or (longitudinal adj (study or studies)).mp. or (cohort adj (study or studies)).mp. or (case control adj (study or studies)).mp. or (follow up adj (study or studies)).mp. or (epidemiologic* adj (study or studies)).mp. or (cross sectional adj (study or studies)).mp. or (survey* or surveillance).mp. or (meta adj analys#s).mp.	917,584
15.	13 or 14	997,671
16.	(polymixinADJ1B or polymixinADJ1 E or colistin or Gentamycine or Amikacine or Tobramycine or streptomycin or ((imipenem adj1 cilastatin adj1 relebactam) or RECARBRIO or MK-7655A)).mp.	99
17.	7 and 16	1
18.	7 and 12 and 15 and 16	0

Table S5. Search strategy- EBMR

#	Query	Results from 18 Feb 2025

1.	exp Gram-Negative Bacterial Infections/ or exp Gram-Negative Bacteria/	12,598
2.	(Gram negative or Gram-negative or GN).ti,ab.	3,386
3.	Drug resistance, microbial/ or Drug resistance, bacterial/ or beta-lactam resistance/ or Drug resistance, multiple, bacterial/ or carbapenem-resistant Enterobacteriaceae/	1,899
4.	(((carbapenem or meropenem or imipenem) adj2 resistan*) or beta-lactamase or carbapenemase or ESBL or ((multi-drug or multi drug) adj2 resistan*) or ((pan-drug or pan drug) adj2 resistan*) or ((extensive* drug or extensive-drug or extensively-drug) adj2 resistan*)).ti,ab.	1,643
5.	1 or 2	15,271
6.	3 or 4	3,377
7.	5 and 6	1,274
8.	exp safety/	5,318
9.	(adverse reaction or safety profile or side effect).mp. [mp=ti, ab, tx, kw, ct, ot, fx, sh, hw]	138,777
10.	8 or 9	143,152
11.	exp Clinical Trial/ or exp Randomised Controlled Trial/ or exp controlled clinical trial/ or multicenter study/ or clinical trial, phase III/ or clinical trial, phase IV/ or clinical trial, phase II/ or random allocation/ or exp Single-Blind Method/ or Double-Blind Method/ or exp Cross-over studies/ or randomi?ed controlled trial$.mp. or rct.mp. or (random$ adj2 allocat$).mp. or single blind$.mp. or double blind$.mp. or ((treble or triple) adj blind$).mp. or exp Prospective Study/	1,166,032
12.	Case-control studies/ or observational study/ or cross-sectional studies/ or health surveys/ or longitudinal studies/ or cohort studies/ or meta-analysis/ or (prospective adj (study or studies)).mp. or (retrospective adj (study or studies)).mp. or (longitudinal adj (study or studies)).mp. or (cohort adj (study or studies)).mp. or (case control adj (study or studies)).mp. or (follow up adj (study or studies)).mp. or (epidemiologic* adj (study or studies)).mp. or (cross sectional adj (study or studies)).mp. or (survey* or surveillance).mp. or (meta adj analys#s).mp.	428,060
13.	11 or 12	1,283,988
14.	(polymixinADJ1B or polymixinADJ1 E or colistin or Gentamycine or Amikacine or Tobramycine or streptomycin or ((imipenem adj1 cilastatin adj1 relebactam) or RECARBRIO or MK-7655A)).mp.	1,468
15.	7 and 14	145
16.	7 and 10 and 13 and 14	22

Figure S1. JBI Risk Assessment for case control studies (N=1)

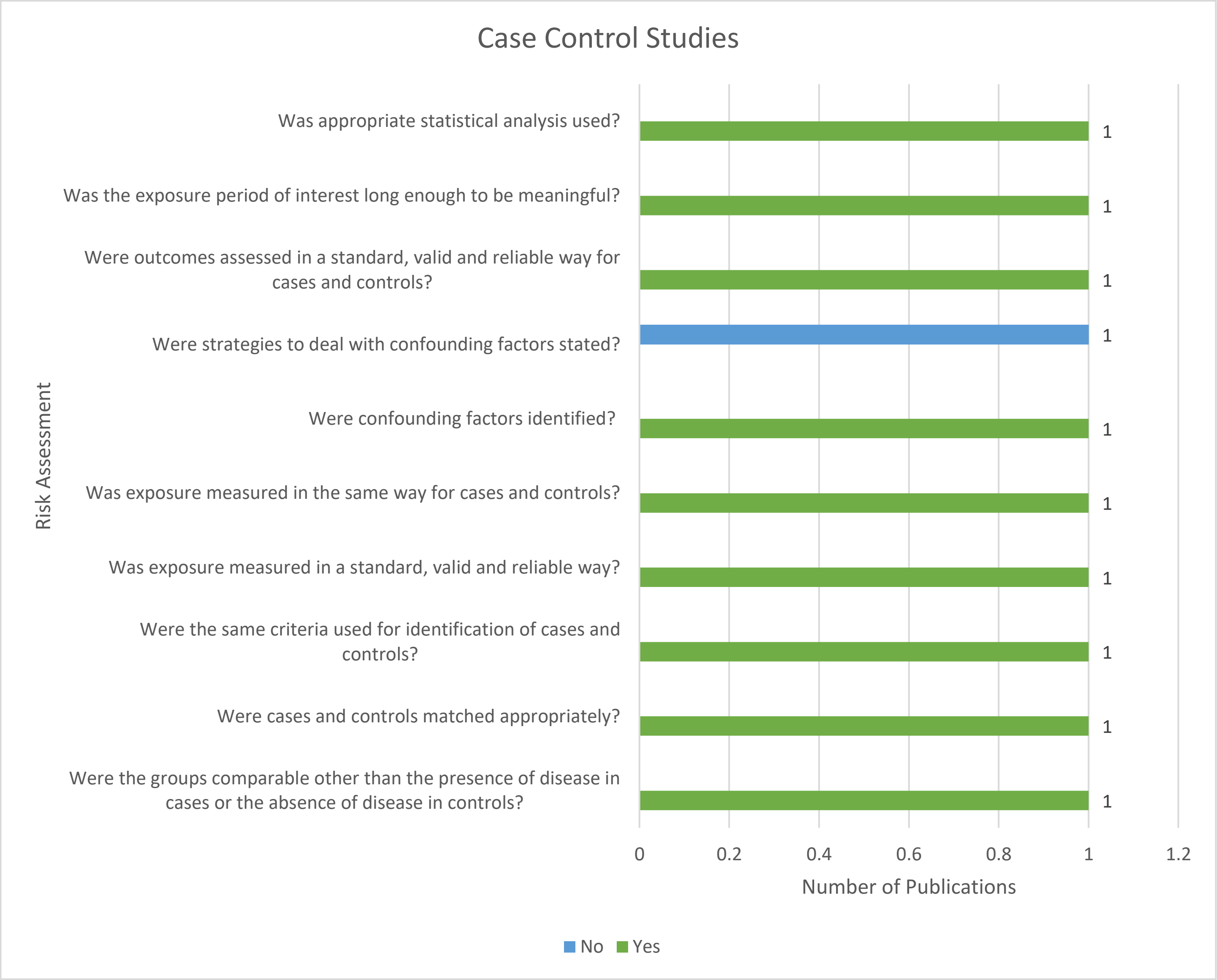

JBI: Joanna Briggs Institute

Figure S2. JBI Risk Assessment for cohort studies (N=48)

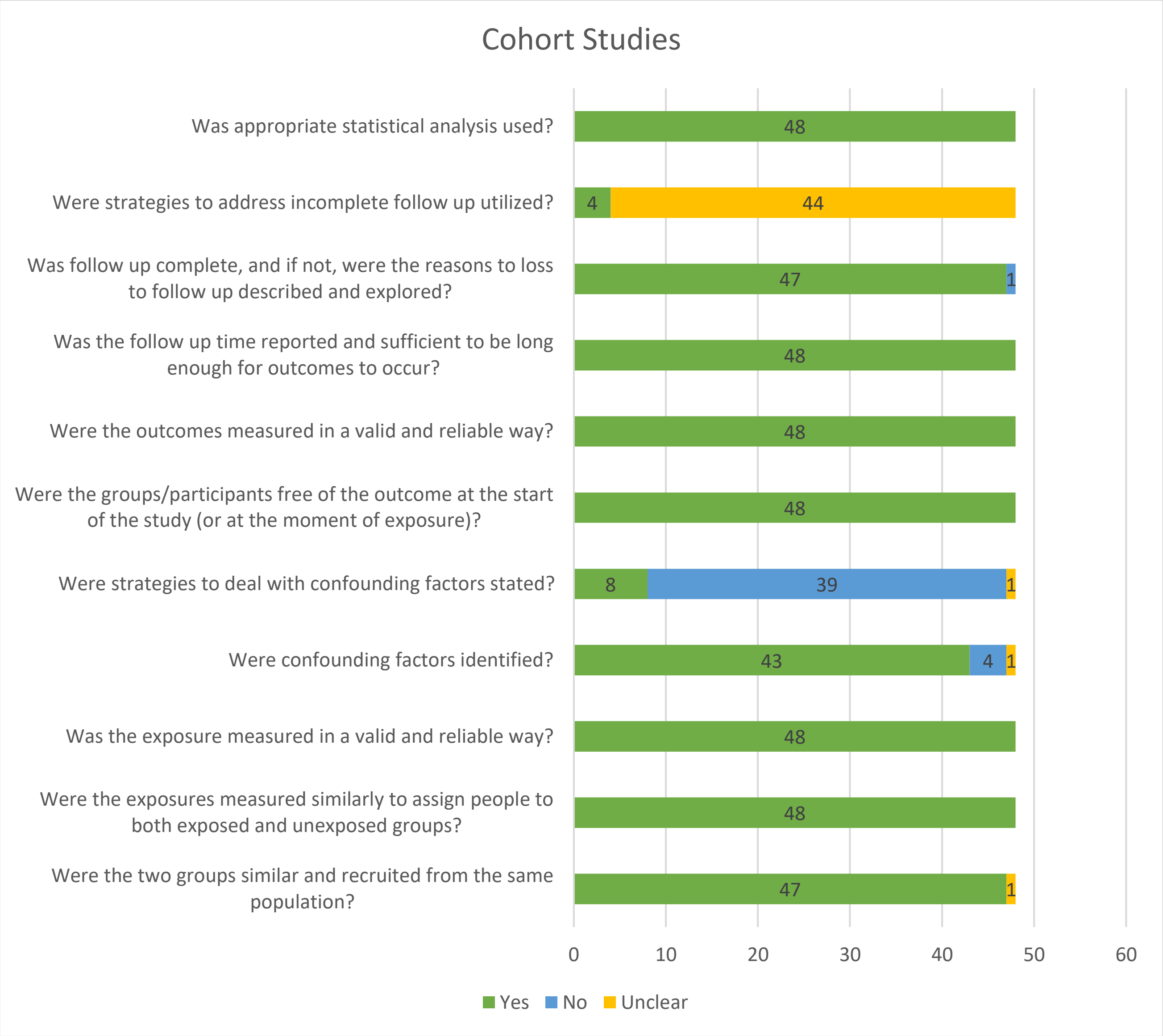

JBI: Joanna Briggs Institute

Figure S3. JBI Risk Assessment for RCTs (N=14)

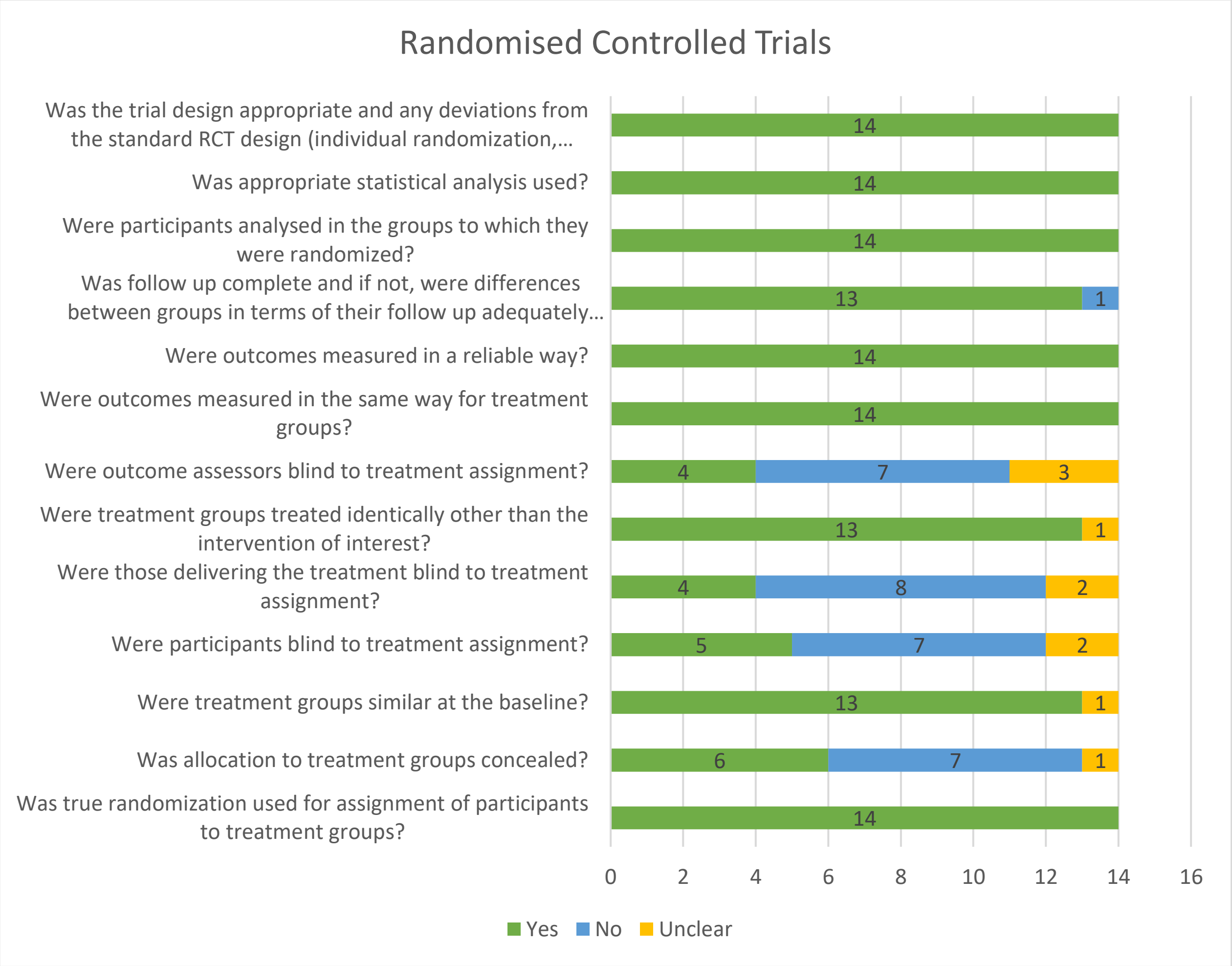

JBI: Joanna Briggs Institute; RCT: Randomised Controlled Trial

Figure S4. JBI Risk Assessment for prevalence studies (N=5)

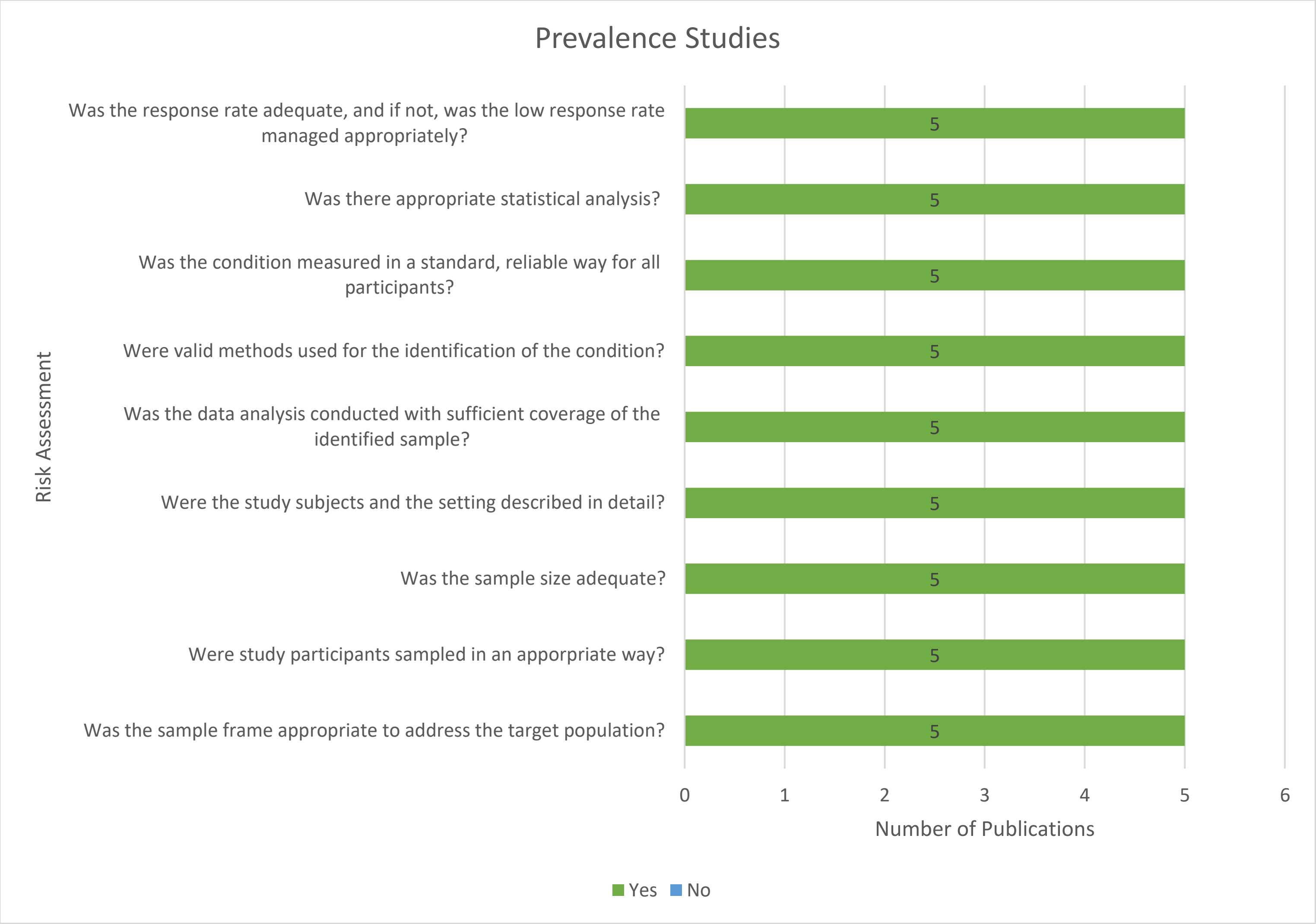

JBI: Joanna Briggs Institute
